# What Should Vaccine Developers Ask? Simulation of the Effectiveness of Malaria Vaccines

**DOI:** 10.1371/journal.pone.0003193

**Published:** 2008-09-11

**Authors:** Melissa A. Penny, Nicolas Maire, Alain Studer, Allan Schapira, Thomas A. Smith

**Affiliations:** 1 Swiss Tropical Institute, Basel, Switzerland; 2 Department of Public Health & Epidemiology, Swiss Tropical Institute, Basel, Switzerland; Federal University of São Paulo, Brazil

## Abstract

**Background:**

A number of different malaria vaccine candidates are currently in pre-clinical or clinical development. Even though they vary greatly in their characteristics, it is unlikely that any of them will provide long-lasting sterilizing immunity against the malaria parasite. There is great uncertainty about what the minimal vaccine profile should be before registration is worthwhile; how to allocate resources between different candidates with different profiles; which candidates to consider combining; and what deployment strategies to consider.

**Methods and Findings:**

We use previously published stochastic simulation models, calibrated against extensive epidemiological data, to make quantitative predictions of the population effects of malaria vaccines on malaria transmission, morbidity and mortality. The models are fitted and simulations obtained via volunteer computing. We consider a range of endemic malaria settings with deployment of vaccines via the Expanded program on immunization (EPI), with and without additional booster doses, and also via 5-yearly mass campaigns for a range of coverages. The simulation scenarios account for the dynamic effects of natural and vaccine induced immunity, for treatment of clinical episodes, and for births, ageing and deaths in the cohort. Simulated pre-erythrocytic vaccines have greatest benefits in low endemic settings (<EIR of 10.5) where between 12% and 14% of all deaths are averted when initial efficacy is 50%. In some high transmission scenarios (>EIR of 84) PEV may lead to increased incidence of severe disease in the long term, if efficacy is moderate to low (<70%). Blood stage vaccines (BSV) are most useful in high transmission settings, and are comparable to PEV for low transmission settings. Combinations of PEV and BSV generally perform little better than the best of the contributing components. A minimum half-life of protection of 2–3 years appears to be a precondition for substantial epidemiological effects. Herd immunity effects can be achieved with even moderately effective (>20%) malaria vaccines (either PEV or BSV) when deployed through mass campaigns targeting all age-groups as well as EPI, and especially if combined with highly efficacious transmission-blocking components.

**Conclusions:**

We present for the first time a stochastic simulation approach to compare likely effects on morbidity, mortality and transmission of a range of malaria vaccines and vaccine combinations in realistic epidemiological and health systems settings. The results raise several issues for vaccine clinical development, in particular appropriateness of vaccine types for different transmission settings; the need to assess transmission to the vector and duration of protection; and the importance of deployment additional to the EPI, which again may make the issue of number of doses required more critical. To test the validity and robustness of our conclusions there is a need for further modeling (and, of course, field research) using alternative formulations for both natural and vaccine induced immunity. Evaluation of alternative deployment strategies outside EPI needs to consider the operational implications of different approaches to mass vaccination.

## Introduction

The demand for an effective vaccine against *Plasmodium falciparum* malaria has stimulated the development of candidates targeted against pre-erythrocytic stages of the parasite, others against blood stages or toxins that cause disease, and yet more against the sexual stages [1]. It is not obvious what level of efficacy needs to be achieved for a malaria vaccine to be worthwhile since even vaccines that only partially protect might offer substantial health benefits, given the enormous burden of *P. falciparum* morbidity and mortality in endemic areas [2], [3]. In fact, the vaccine that is most advanced in clinical development, RTS,S, has shown only partial protection against infection and disease in clinical trials [4], [5].

The impact of a vaccine will depend not only on average efficacy, but also on the extent of heterogeneity of the host response including its duration. Other determinants include the natural force of infection and its seasonal variation, the vaccination coverage which could be achieved, especially in the most exposed and the most vulnerable groups and the efficacy and coverage of other malaria control interventions, preventive or curative. As for all public health interventions, safety, cost, operational feasibility and acceptability also need to be considered when deciding which candidates to prioritize, which ones to consider for combination, and which ones to develop for specific target groups or deployment strategies.

Field trials of malaria vaccines are generally designed to evaluate the effect on morbidity or on infection rates in the vaccinated population [6], without considering effects on transmission or the long-term dynamics of immunity. In contrast, modeling of malaria vaccines has concentrated on analysing transmission, and in particular identifying the conditions for controlling or interrupting it [7]–[11]. We have developed stochastic simulation models of the natural history and epidemiology of *P. falciparum* malaria [12], [13] that bring all these factors together and apply them to the simulation of malaria vaccination [14]. In a first phase of the project we concentrated on the likely epidemiological effects [14] and cost-effectiveness [15] of pre-erythrocytic vaccines when delivered in areas of stable malaria via the Expanded Program on Immunization (EPI). We now report on the extension of these simulations to consider also blood-stage vaccines, mosquito stage transmission blocking vaccines and combination vaccines, delivered via different modalities. The purpose of this work is to assess the effectiveness of different vaccines at different transmission settings, to examine what minimal profile of a vaccine is appropriate and to prompt discussion of alternative delivery modalities.

## Results

### Effects on transmission

**Figure 1:**
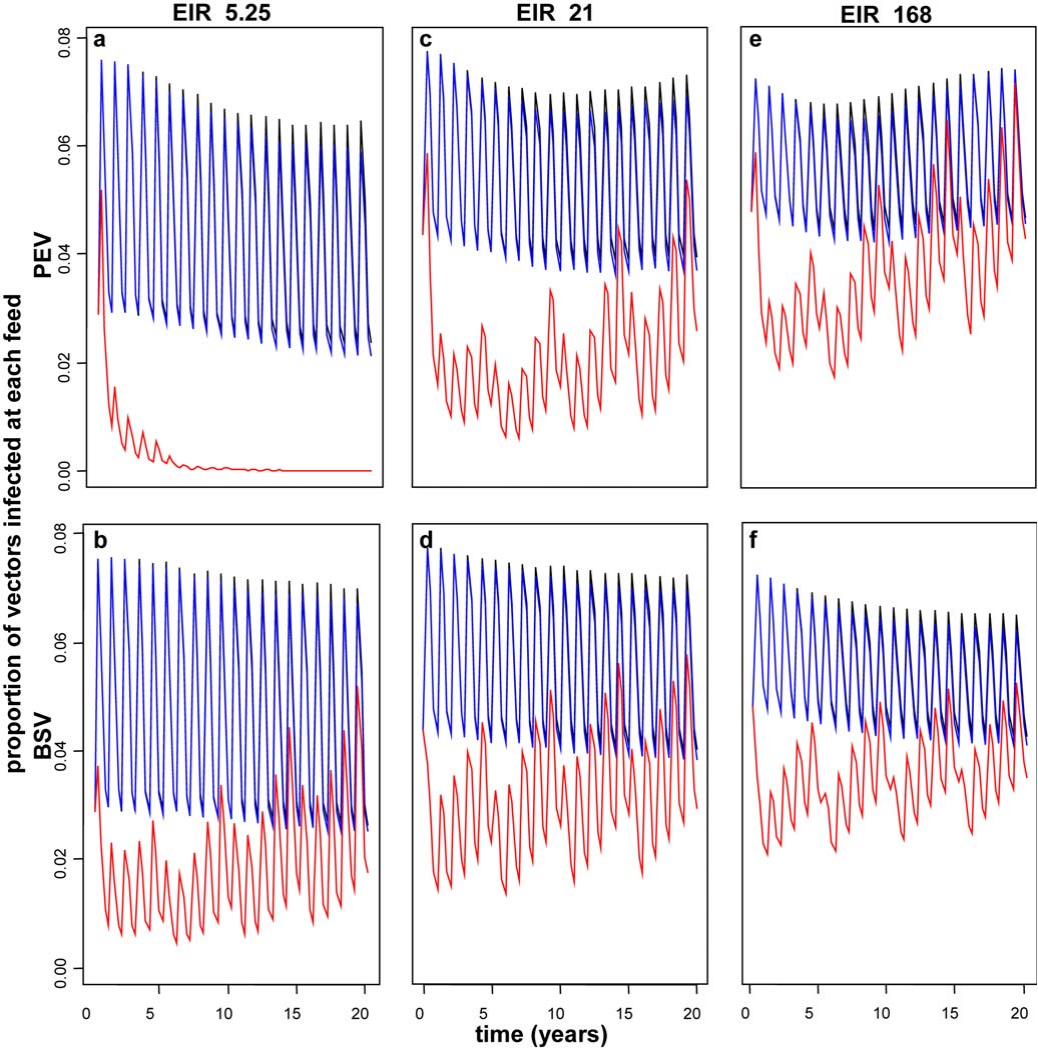
Effect of PEV (a,c,e) and BSV (b,d,f) on infectivity to vector over 20 years when delivered via EPI (black), EPI with boosters (blue) and EPI with 95% mass vaccination (red) for transmissions settings of EIR 5.25 (a,b), 21 (c,d) and 168 (e,f). Results obtained assuming a vaccine efficacy of 80%, half-life of 10 years and homogeneity value of 10. Note that the blue and black lines almost overlap.

In line with our previous simulations, we find that moderately efficacious pre-erythrocytic vaccines applied via EPI do not have any substantial effect on malaria transmission, (results not shown), because only a small proportion of the population is protected. If the initial efficacy of PEV is high then effects on transmission are observed for EPI and EPI with boosters (Figure 1a, c, e). If a high efficacy PEV is delivered via EPI with mass vaccination with high coverage, herd immunity with substantial transmission effects are achieved (Figure 1a, c, e).

We observe elimination with PEV alone, at very high efficacy and mass vaccination coverage and at the lowest transmission levels (Figure 1a), where PEV is more effective than BSV (see below). However, these models do not consider immigration and importation of new cases, which is far removed from any real life epidemiological situation. In other PEV scenarios where we observe some effect on transmission without interruption, the reduction is followed by rebounds in infectivity (Figure 1a, c, e). This is a consequence of the deferral of infection events for such a vaccine. Reduction in incidence of blood stage infection for younger hosts makes them vulnerable to high-density parasitaemia, which is most infectious, when they are older. The simulation assumes mosquitoes bite these larger hosts more frequently [19], thus leading to a increase in transmission.

For highly efficacious BSV, we observe effects on transmission, particularly at high transmission settings (Figure 1b, d, f). These reductions are slightly less than those observed for PEV with the same initial efficacy, and BSV does not interrupt transmission in any scenario. Rebound effects on infectivity occur for BSV only when delivered via mass vaccination (Figure 1 b, d, f). Combinations of PEV and BSV produce reductions in transmission slightly greater than the individual vaccines alone, and at higher transmission a rebound occurs, as for PEV alone (results not shown).

**Figure 2:**
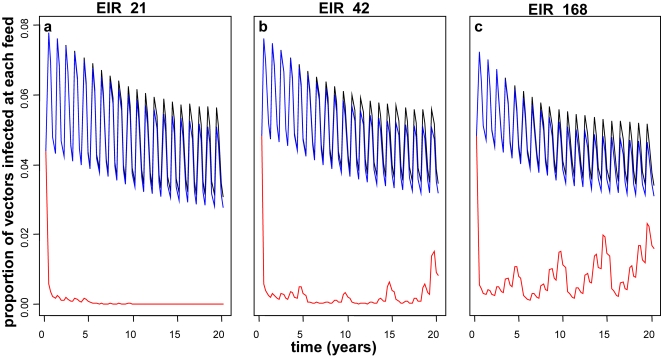
Effect of PEV with MSTBV (a,b,c) on infectivity to vector over 20 years when delivered via EPI (black), EPI with boosters (blue) and EPI with 95% mass vaccination (red) for transmissions settings of EIR 21 (a), 42 (b) and 168 (c). Results obtained assuming a vaccine efficacy of 52%, half-life of 10 years and homogeneity value of 10.

As expected, for vaccine combinations with MSTBV we observe greater reductions in transmission over PEV or BSV alone (Figure 2 for PEV with MSTBV, other results not shown). In contrast to PEV alone, we observe no rebound in infectivity for delivery modes EPI and EPI with boosters and over 20 years, MSTBV combination vaccines delivered via EPI with boosters reduce transmission to a slightly greater extent than in the absence of the boosters. Under EPI with mass vaccination at 95% coverage, MSTBV combinations reduce transmission to zero at low transmission. In higher transmission settings we observe substantial effects on transmission and then periodical rebounds over 20 years (Figure 2).

Elimination is generally simulated at the lower initial transmission intensities with vaccine combinations containing MSTBV and/or for highly efficacious vaccines delivered via EPI with mass vaccination. In the simulations that we examined in detail (results not shown) elimination is more likely when the homogeneity parameter is very low or if the vaccine half-life is very large.

**Figure 3:**
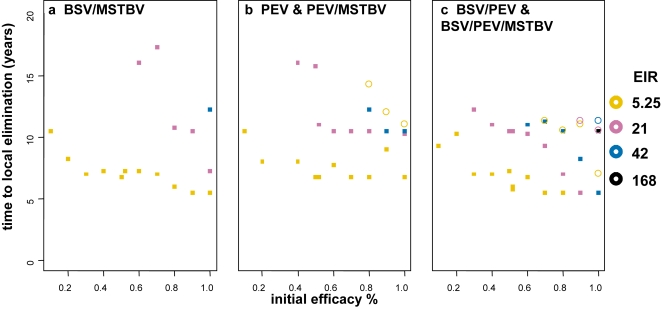
Time to elimination given initial efficacies (x-axis) of vaccine for different transmission settings (square indicates combination with MSTBV and circle without). All results are for vaccines delivered via EPI with mass vaccination, no elimination is achieved under these conditions for vaccines delivered via EPI or EPI with boosters. Results obtained assuming vaccine half-life of 10 years and homogeneity value of 10.

The time to elimination, dependent on initial vaccine efficacy, is considered in Figure 3, for those transmission settings and delivery modalities (mass vaccination at 95% coverage) where elimination was achieved within 20 years. Here, each vaccine has a half-life of 10 years and homogeneity value of 10. Combinations with MSTBV achieve elimination for lower initial efficacies of the other vaccine component and for a wider range of transmission settings than PEV or PEV with BSV. Interpretation of the time to elimination given the vaccine profile is difficult because of the discrete nature of the mass vaccination campaigns. In general, the time to elimination given this mass vaccination schedule does not strongly depend on the initial efficacy except at very high efficacy levels. In the lowest transmission settings with very high coverage and high initial efficacy of the MSTBV component, elimination is observed even with very low initial efficacies of the other components.

### Effects on morbidity and mortality

#### Pre-erythrocytic vaccines

**Figure 4:**
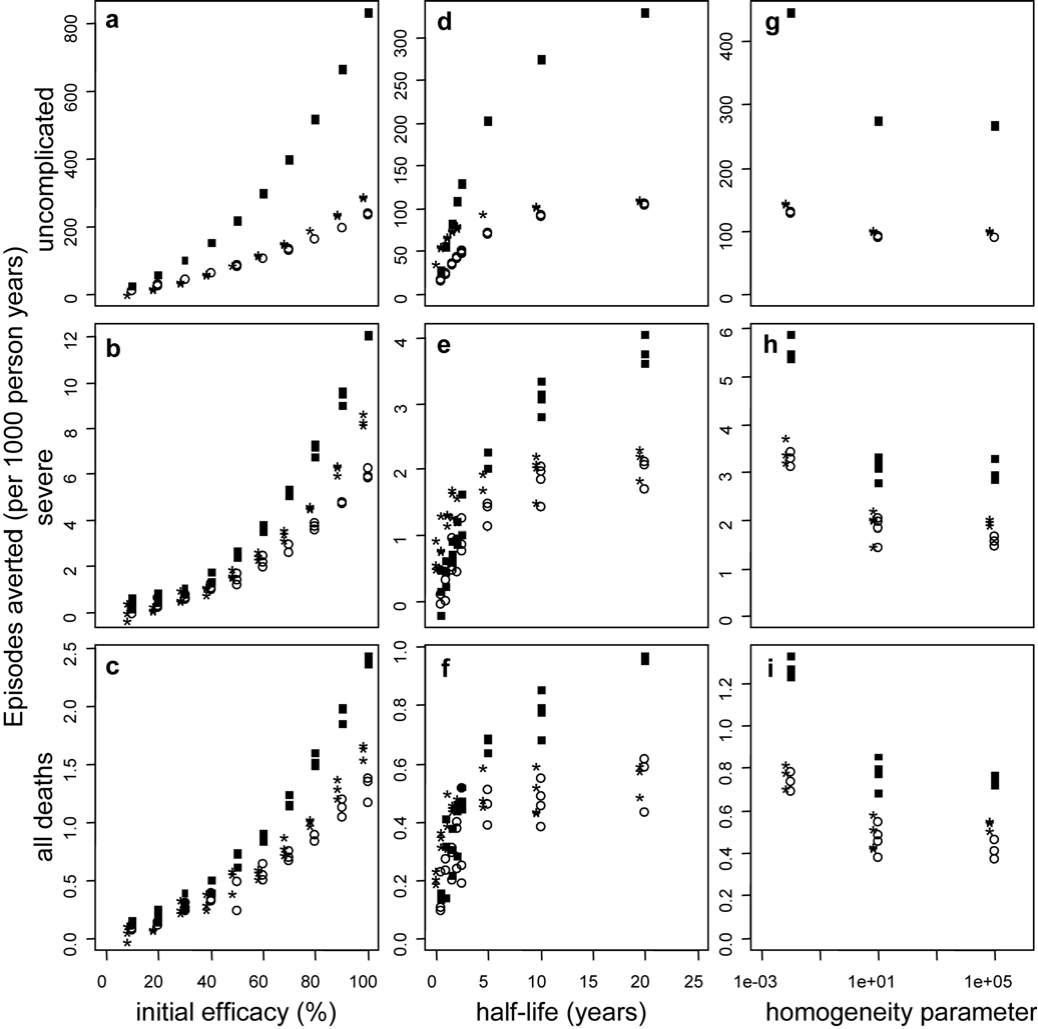
Effect of initial efficacy (a–c), vaccine half-life (d–f) and degree of heterogeneity (g–i) on the number of events averted per 1000 person years by PEV for the reference transmission setting of EIR 21. Results obtained assuming vaccine efficacy of 52%, a vaccine half-life of 10 years and homogeneity value of 10, unless the values are varied along the x-axis. Vaccines are distributed via EPI (circles), EPI with boosters (*) and EPI with 70% mass vaccination (squares).

**Figure 5:**
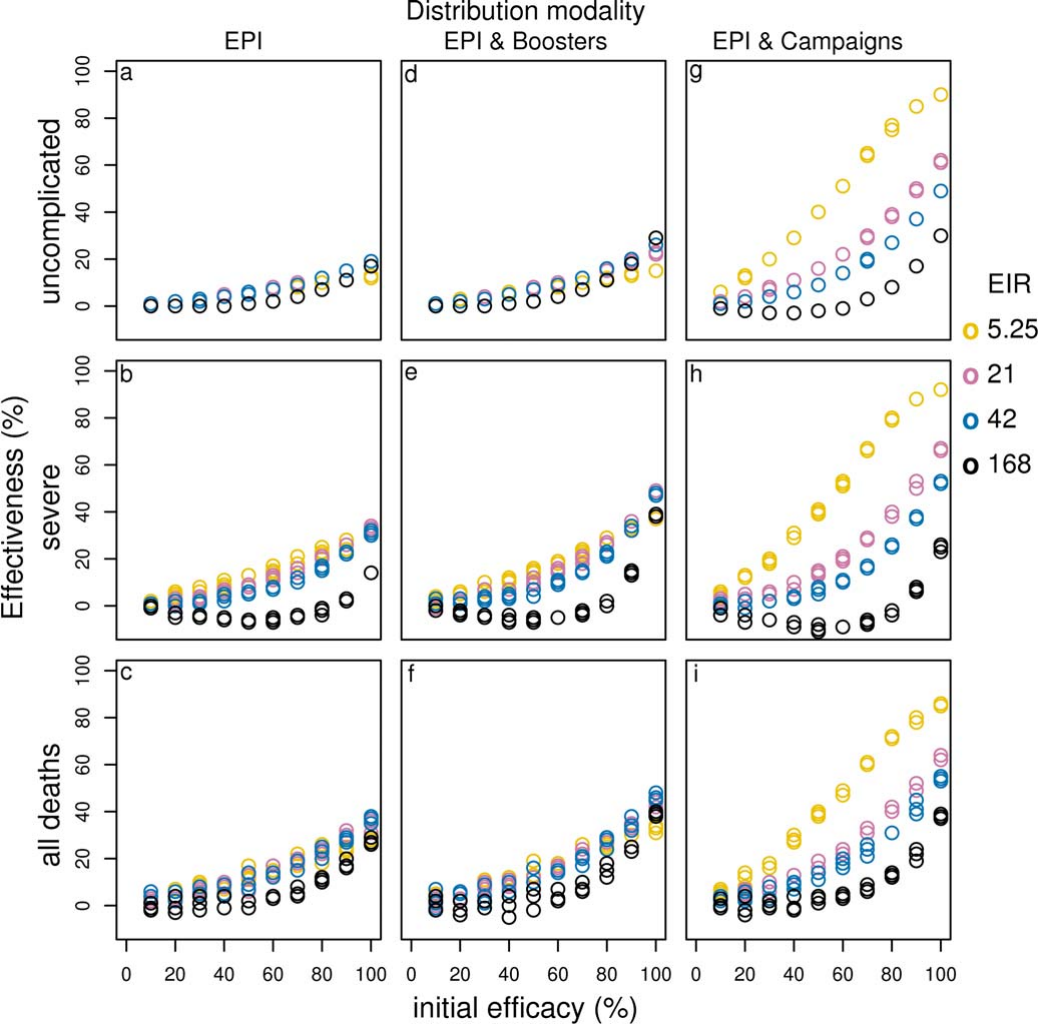
Effect of initial efficacy on effectiveness of PEV for different transmission settings delivered via EPI (a–c), EPI with boosters (d–f) and EPI with 70% mass vaccination (g–i). Results obtained assuming a vaccine half-life of 10 years and homogeneity value of 10.

*Effect of vaccine characteristics:* The numbers of events averted per 1000 person-years by a pre-erythrocytic vaccine, when distributed via EPI (Figure 4a–c at the reference transmission setting, seasonal transmission based on Namawala, Tanzania) are similar to those reported in our previous analyses [14] despite the small changes to the health system and the epidemiological model. Even moderately efficacious PEVs delivered via EPI may avert substantial numbers of clinical events (Figure 4a–c and Table S1, Supplementary material). The curves relating effectiveness to vaccine efficacy for a range of transmission settings, (Figure 5) are concave, indicating that an increase in efficacy has a greater than proportional effect on events averted, due to the impact on transmission.

**Figure 6:**
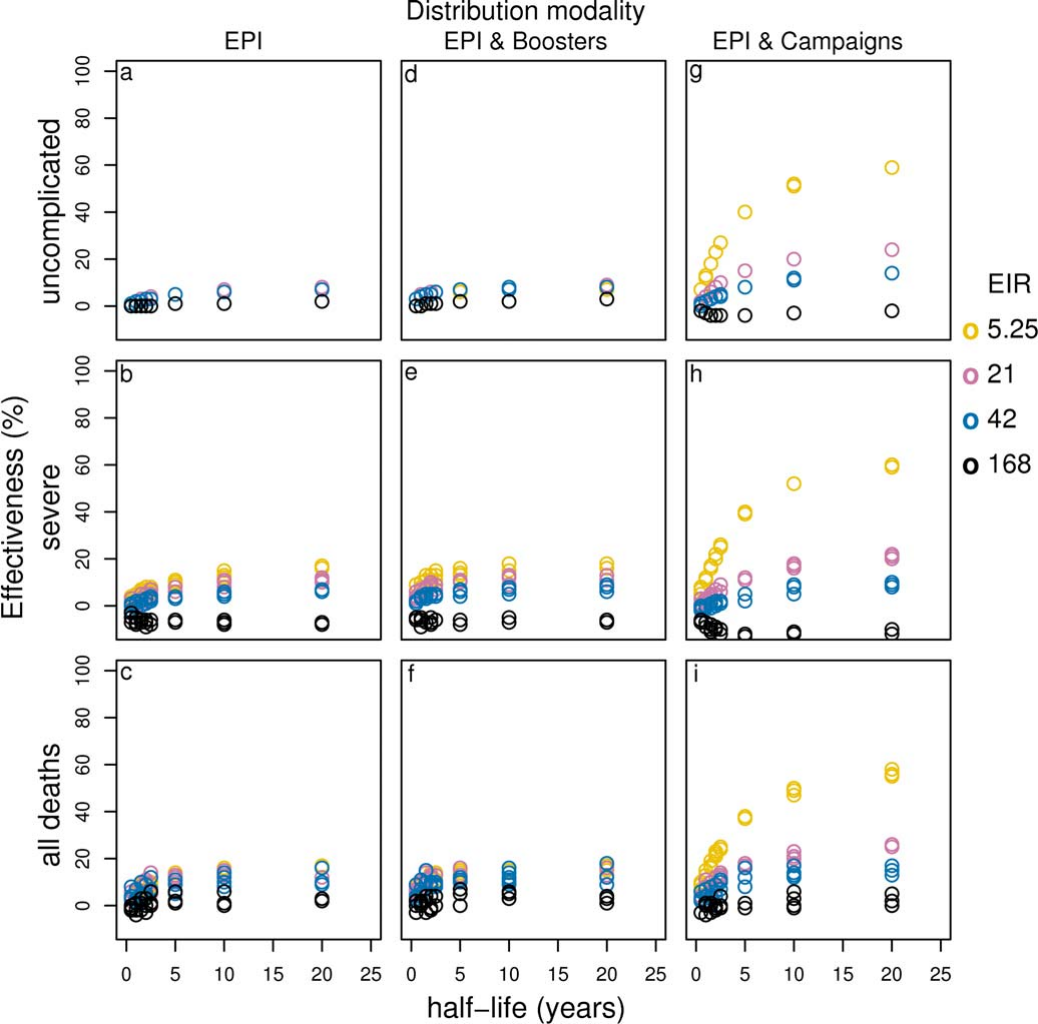
Effect of vaccine half-life on effectiveness of PEV for different transmission settings delivered via EPI (a–c), EPI with boosters (d–f) and EPI with 70% mass vaccination (g–i). Results obtained assuming a an initial vaccine efficacy of 52% and homogeneity value of 10.

As reported previously, the effectiveness of PEV depends strongly on the duration of protection for vaccines with half-life less than 2–3 years [14] deployed by EPI or EPI with boosters. However, there is only a modest increase in the number of disease episodes or deaths averted, if the half-life is extended from 2 years to 5 years (Figure 4d–f, Figure S1d–f, Figure 6d–f).

Previous simulations showed that a PEV averts a higher proportion of clinical episodes and deaths if there is heterogeneity in the response to vaccination among individuals, namely if PEV concentrates its effects in some individuals (low value for the homogeneity parameter), who thus never become infected, than one that spreads protection more evenly across the population. This result is confirmed here (Figure 4g–i and Figure S1g–i), and the effect, although small, is observed over all transmission settings (results not shown) and is particularly true in mass vaccination scenarios for low transmission settings.

*Effect of duration of observation and transmission intensity:* By using a 10 year period we obtain slightly more favourable predictions than found previously for a 20 year horizon, since effects of deferral of episodes become most evident in the second decade after the start of the program [14]. This is particularly the case for higher transmission settings (results not shown) and for severe malaria episodes. As found previously, [14] at the reference transmission setting of EIR of 21, the percentage of deaths averted remains fairly constant over the course of the follow-up, and a PEV of 50% primary efficacy is predicted to avert about 13% of all deaths under EPI alone, about 15% under EPI with boosters and about 18% and 21% with EPI and mass vaccination of coverage 50% and 70%, respectively (Fig. S2, Supplementary material). For all vaccine distribution systems, the proportion of uncomplicated episodes that are averted in this transmission setting is lower than for deaths and severe episodes (Figure S1a–c).

*Effect of different delivery modalities:* Across all transmission settings, the addition of booster doses to the EPI vaccine schedule results in minimal improvement to the cases averted by the reference PEV (Figure 4, Figure S1, Figure 5a–f). However a benefit of booster doses is evident when the half-life is short. Dissemination of vaccines via mass campaigns, supplementing EPI, also has little impact on overall effect when the efficacy is low, but at low transmission, high efficacy and high vaccine coverage, when elimination is achieved, all but the earliest episodes are averted. There is also a substantial benefit of mass vaccination in medium transmission settings, particularly for measures of morbidity (Figure 4, Figure 5). In contrast, at very high transmission settings, mass vaccination averts fewer cases than EPI or EPI plus booster delivery, especially at low vaccine efficacies and for severe episodes. This is because PE vaccines delay infections for the whole population under high and moderate mass vaccination coverage (Supplementary Figure S2). This is also true for very low vaccine half-life (results not shown).

#### Blood-stage vaccines

**Figure 7:**
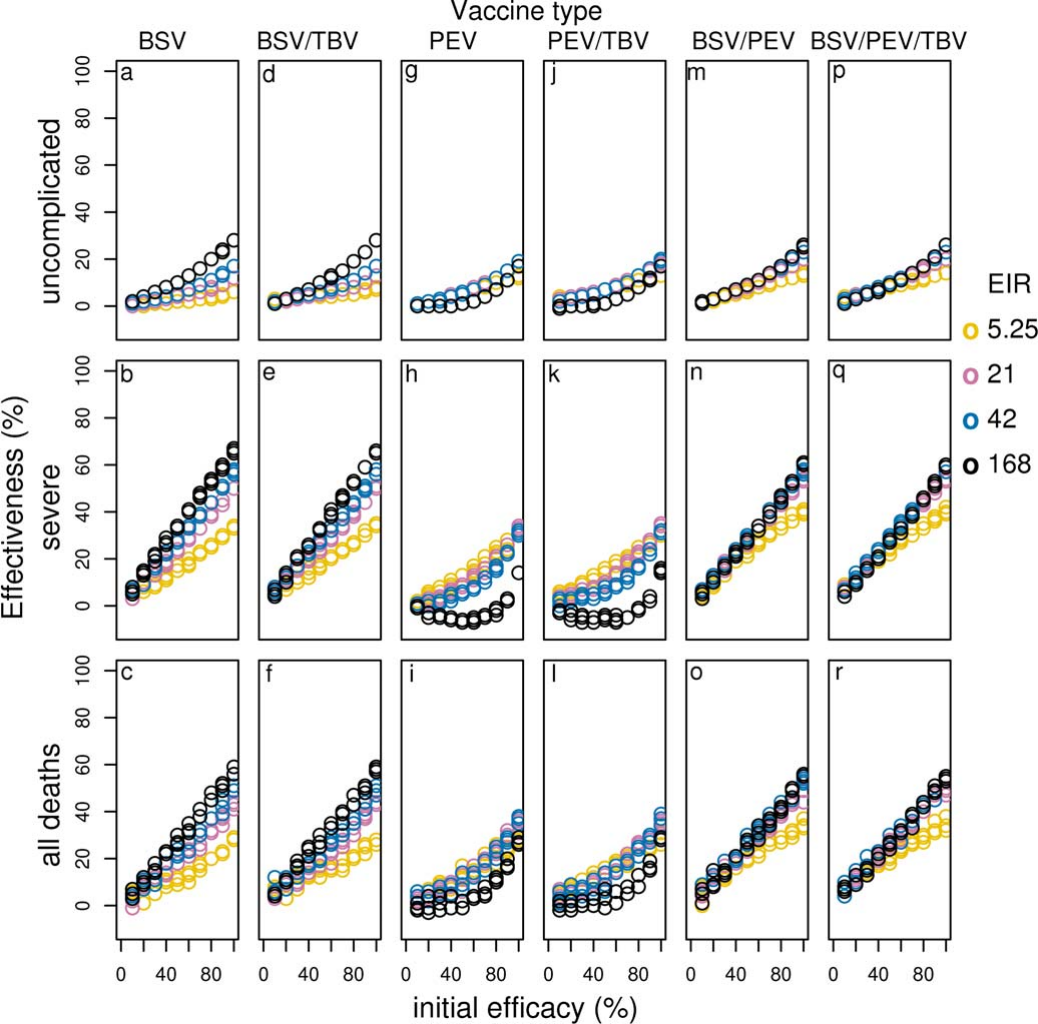
Effect of initial efficacy on effectiveness of all vaccines for different transmission settings delivered via EPI (BSV (a–c), BSV/TBV (d–f), PEV (g–i), PEV/TBV (j–l), BSV/PEV (m–o) and BSV/TBV (p–r)). Results obtained assuming a vaccine half-life of 10 years and homogeneity value of 10.

*Effect of vaccine characteristics and transmission intensity:* In low transmission settings, low or moderately efficacious blood-stage vaccines avert comparable proportions of disease episodes and deaths to those averted by PEV with comparable efficacy for all delivery modalities. However, at moderate to high efficacy levels, BSVs avert slightly less uncomplicated cases than PEV, but slightly more severe episodes and deaths (compare Figures 5 and S3 and Figures 7, S4, 8a–c, g–i). In transmission settings comparable to the reference level or higher, BSV of low to moderate efficacies avert higher numbers of cases and deaths than PEV. (Figure 7, S4, 8 a–c, g–i).

**Figure 8:**
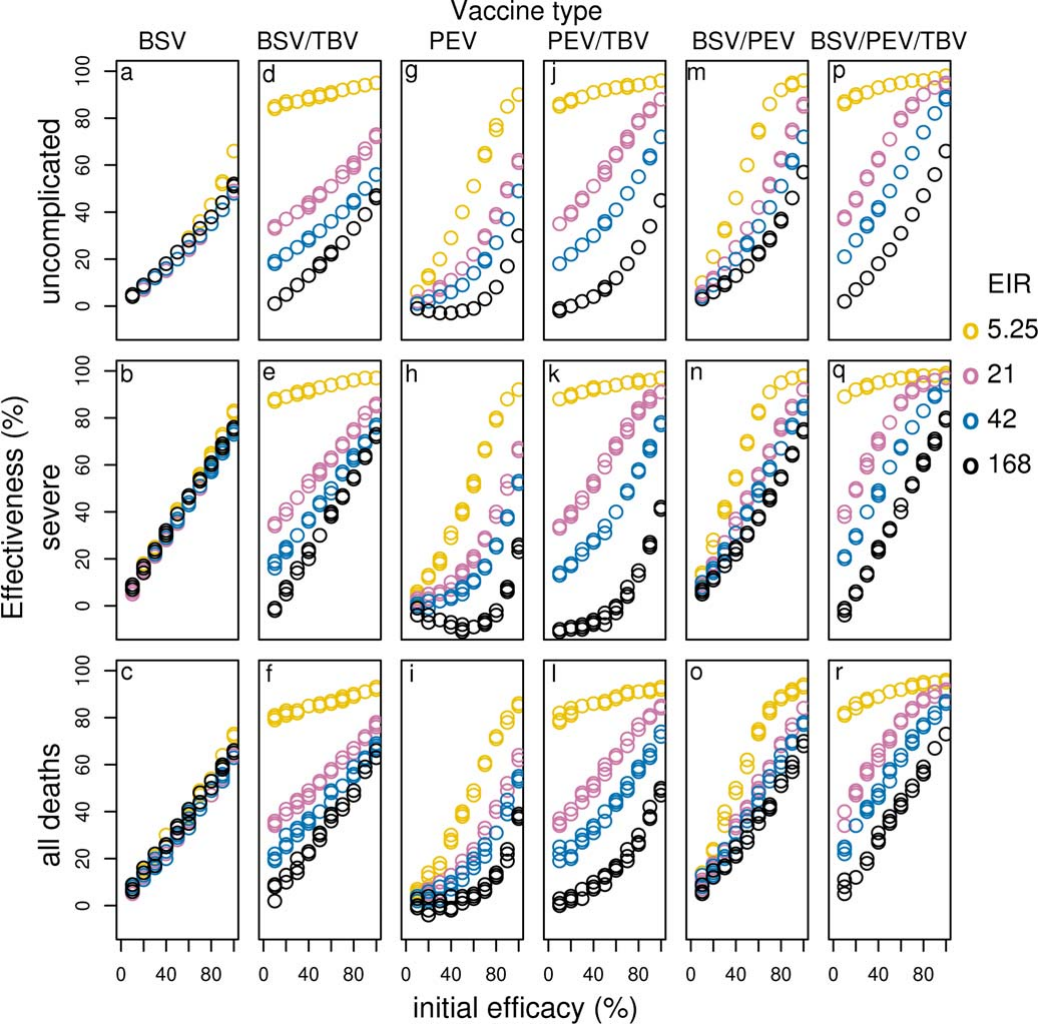
Effect of initial efficacy on effectiveness of all vaccines for different transmission settings delivered via EPI with 70-% mass vaccination (BSV (a–c), BSV/TBV (d–f), PEV (g–i), PEV/TBV (j–l), BSV/PEV (m–o) and BSV/TBV (p–r)). Results obtained assuming a vaccine half-life of 10 years and homogeneity value of 10.

The curves relating the events averted (not shown) and effectiveness of BSVs to the primary efficacy (Figure S3) are close to straight lines, indicating that the effectiveness is almost directly proportional to the efficacy. Probably, the effect on transmission of highly efficacious BSV, (Figure 1b, d, f) is less important for averting disease and death than is the case for PEV.

As with PEV, the effectiveness of BSV strongly depends on the duration of protection for vaccines with half-life less than about 2–5 years (Figure S5a–c, Figure S6a–c), while increasing the half-life beyond 5 years has little impact.

As with PEV, the results predict a very small improvement if the vaccine effect is concentrated in some individuals. (Figure S7 a–c).

*Effect of different delivery modalities:* The addition of boosters to the EPI for all transmission settings has minimal effect on the effectiveness of BSV, though we observe small gains by adding boosters to the EPI at very high vaccine efficacies, or short half-lives. In general, the effect of adding mass vaccination is much greater (Figure 7, S4 and 8a–c).

#### Combination vaccines and MSTBV

*BSV plus PEV: C*ombining BSV with PEV improves effectiveness over PEV alone for all transmission settings and vaccine deliveries (compare Figures 7, S4, 8 m–o with g–i and Supplementary Table S1). The greatest difference is seen at high transmission, and this can be attributed to the high effectiveness of BSV alone in these settings. In fact, in high transmission settings, the combination of PEV with BSV is less effective than BSV alone due to the poor effectiveness of PEV at such transmission settings. In contrast, the combination is more effective than BSV alone in low transmission settings (compare Figures 7, S4, 8 a–c and g–i with m–o and Supplementary Table S1).

*MSTBV:* MSTBV show minimal effectiveness when used alone, except when delivered via mass vaccination, at very high coverage and efficacy (Figures 7, S4, 8 d–f, j–l, p–r and Figure S8 at the 0 initial efficacy point for the other vaccines in the combinations). Combinations of MSTBV with PEV or BSV do not appear to improve the effectiveness of the vaccines alone when delivered via EPI or EPI with boosters over 10 years (Figures 7 and S4 compare d–f with a–c and j–l with g–i), and over 20 years only slightly more events are averted (results not shown). There is also little to be gained by triple combination delivered through EPI (Figures 7 and S4, compare p–r with m–o). In contrast, with mass vaccination, combinations with MSTBV are much more effective (Figure 8). This is the case for all transmission settings and for all events averted, except in the case of highly efficacious BSV combined with MSTBV at very high transmission. In such a scenario the effectiveness of BSV is slightly higher than for BSV combined with MSTBV for severe disease and as coverage of the mass campaign decreases, this effect is also seen for uncomplicated cases. This is because MSTBV blocks transmission and hence natural immunity development and thus, in the absence of elimination, delays severe disease even with BSV protection.

The biggest improvement to effectiveness by adding MSTBV to BSV, PEV or BSV with PEV, is observed at very low transmission settings, where almost all deaths, severe and uncomplicated events tend to a situation where they may all be averted over the 10 years for very high efficacy and mass vaccination coverages, suggesting local elimination could be achieved under such “ideal” conditions. (Figure S8).

The results indicating significant improvement to the number of cases averted for vaccine combinations with MSTBV is achieved only when delivered via EPI with mass vaccination (Figure 8), are not surprising when one considers that delivery of vaccines via EPI and EPI with boosters target only infants and children up to four years and that once a child misses an EPI dose they do not receive any further doses. In such situations the effect of MSTBV is concentrated in age groups of the population that contribute little to transmission. Over a 20 year time period, we do observe an increase in effectiveness by combination vaccines with MSTBV, compared to those without, especially with increasing half-life (results not shown).

With mass vaccination, the effectiveness of combination vaccines continues to increase with duration of protection even for half-lives beyond 5 years, in contrast to other delivery modalities (compare Figure S6 d–f, j–l, q–s). Additionally, for combinations with MSTBV at high coverage levels in low to moderate transmission settings elimination is approached, or achieved depending on the coverage level (Figure S6). Like for single vaccines, a high degree of heterogeneity improves effectiveness, but this effect is far more pronounced for combinations (Figure S7).

#### Effect of delivery modality and coverage in general

In most contexts, EPI with booster does not significantly improve cumulative effectiveness or cases averted over EPI alone. In contrast, mass campaigns increase effectiveness even at relatively low coverage, especially in low transmission settings. However, in high transmission settings the increase in effectiveness of delivery via EPI with mass vaccination is not as large. For very high transmission, a PEV with low efficacy is less effective under EPI with mass vaccination compared to EPI alone (compare Figure 5 a–c with g–i and Supplementary Table S1).

**Figure 9:**
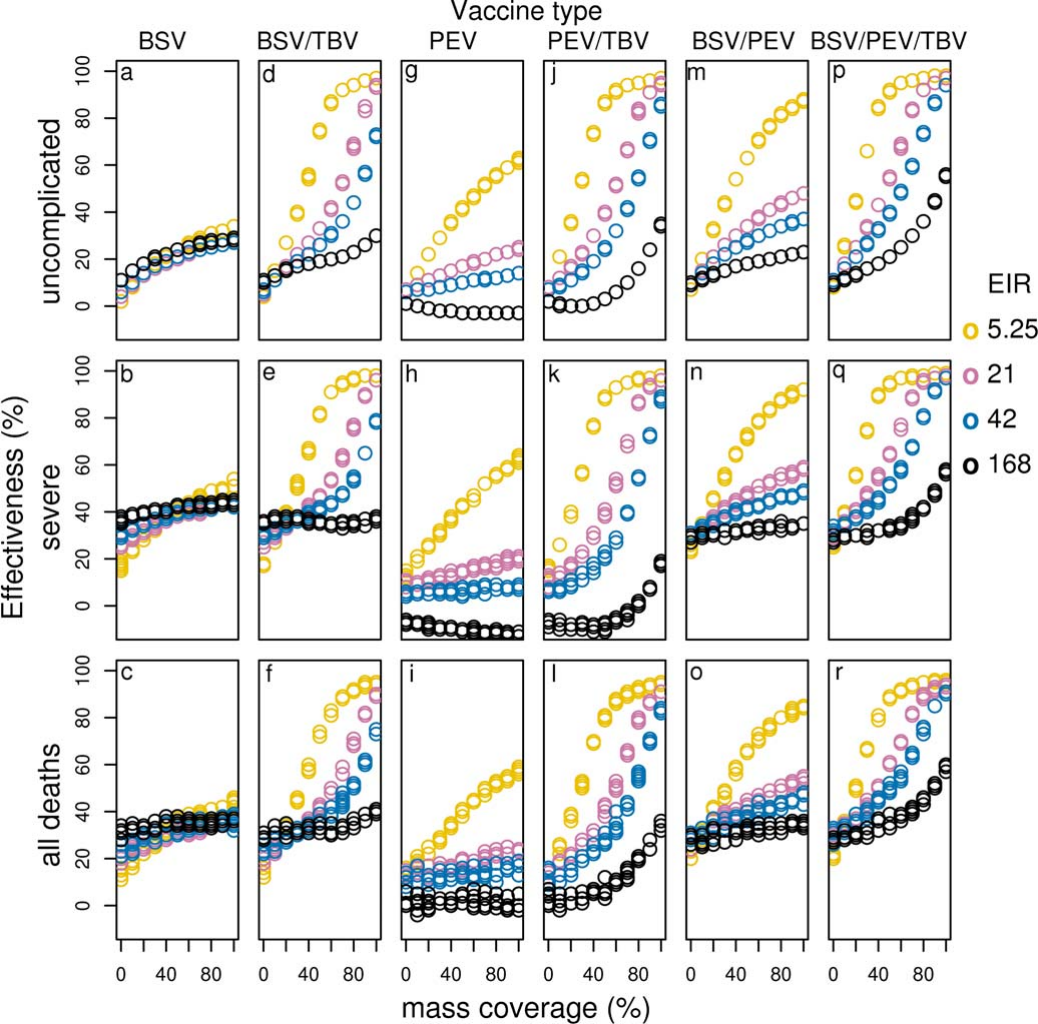
Effectiveness of vaccines given different levels of mass vaccination coverage when delivered via EPI with community wide campaigns for different transmission settings (BSV (a–c), BSV/TBV (d–f), PEV (g–i), PEV/TBV (j–l), BSV/PEV (m–o) and BSV/TBV (p–r)). Results obtained assuming an initial vaccine efficacy of 52%, a vaccine half-life of 10 years and homogeneity value of 10.

Under EPI with mass vaccination, increasing coverage increases effectiveness and cases averted in most transmission scenarios. The exceptions are in higher transmission settings for severe episodes averted by BSV with MSTBV, PEV alone and PEV with MSTBV, where very high coverage levels are associated with small reductions of effectiveness (Figure 9). Although higher coverage of a mass vaccination campaign generally predicts more benefits in terms of episodes averted, a cost effectiveness analysis will offer more insights on what constitutes a feasible level of coverage.

## Discussion

We have presented the results of stochastic simulations of the likely population effects of different malaria vaccine profiles, distributed via different distribution modalities with the aim to assess their usefulness in multiple transmission settings. We examine the effects in controlling morbidity and mortality as well as impact on malaria transmission, thus bridging a gap between field trials of efficacy and malaria transmission models. The simulation results have implication for vaccine developers concerning which vaccines to develop and with what minimal profile, and in which transmission settings these vaccines are likely to provide most benefit. The results highlight the benefit of considering alternative methods of deployment outside of EPI. All these considerations need to be considered by vaccine developers along with issues of safety, immunogenicity, feasibility, and costs that fall outside the scope of this analysis.

Our previous simulations of PEV, aligned with the results of field trials of the RTS,S/AS02A vaccine [18], suggest that such vaccines distributed via EPI will lead to partial protection broadly distributed across the vaccinated population but that no-one is completely protected, and little herd immunity [18]. The new simulations indicate that major gains in effectiveness are unlikely with such vaccines, even if deployed via mass vaccination strategies. In contrast, PEV with higher initial efficacies (70% or more) are predicted to have important herd immunity effects in all delivery modes, especially in mass vaccination situations and in low transmission settings. However, over longer time periods a rebound in infectivity and transmission is expected, and thus fewer cases would be averted, due to deferral of events into older, more infectious, hosts.

The simulations suggest that PEV in general will be much more effective in low transmission settings than in high transmission settings. If delivered via EPI with mass vaccination in high transmission settings and high coverage our models predict that low efficacy PEV may even lead to increases in severe morbidity over a 10 year period (or longer) by shifting the morbidity patterns to those observed in lower exposure settings, i.e. higher risk in higher age-groups. This predicted negative benefit of low efficacious PEV highlights a possible risk involved with introducing PEV in high transmission settings.

The model for the action of asexual BSV represents one of the more tentative components of our integrated model. Although one candidate BSV has shown a substantial effect on parasite densities [20], the assumption that this effect is a constant over different parasite densities in different epidemiological contexts is clearly a simplification. Very likely, effective BSV has complex effects on parasite dynamics, and improved within-host models are needed to capture these. Such a model is currently under development. With this note of caution, the simulations also support the development of BSV candidates as morbidity and mortality control tools, but imply that these are unlikely to have very substantial effects on transmission, unless they achieve very high levels of efficacy in controlling parasite densities, and/or if deployed via mass vaccination. In contrast to PEV, BSV deployed via EPI are predicted to be more effective in higher transmission settings than at low transmission. The converse was observed for BSV delivered via EPI with mass vaccination.

More generally, there is a need for further alternative simulations of both natural and vaccine induced immunity, including models of natural boosting. When field data become available they may require us to consider exposure-dependence of vaccine efficacy or intrinsic age dependence of the primary effect of the vaccine.

In comparing different vaccine types it is important to remember that the measure of efficacy used to define the simulated vaccines differs for the different vaccine types; a 50% efficacy BSV is equivalent to a 50% efficacy PEV only in the sense that both represent imperfect vaccines. The present simulations of combination vaccines, with matched values of the efficacy parameters for the PEV and BSV components represent only one of an infinite number of possible combinations, and it will be most useful to simulate actual candidates, once their likely profiles become available. It seems unlikely that there would be much interest in combining very efficacious PEV or BSV with a rather poor efficacy partner. Combination vaccines with PEV and BSV components seem to have some potential in mass vaccination scenarios, even when efficacy is modest, but do not look much more promising for use in the context of EPI or EPI with boosters than the best of the individual components. In general, the effectiveness of such combinations seems similar to or lower than that of BSV in high transmission settings. This could be attributed to the PEV component lowering the exposure of vaccinated individuals so that the combination vaccine effectiveness is similar to that at a slightly lower transmission level for BSV alone (see Figure 7). At lower levels of transmission the addition of BSV to PEV results in small gains with slightly more events averted than that of PEV alone. However, in practice, the difficulties of epidemiological stratification, seasonal and epidemic variation and the variable effects of vector control might make the use of such combinations rational.

The most realistic scenarios for MSTBV however are clearly situations where high efficacy MSTBV might be deployed in mass vaccination to supplement the effects of moderate efficacy PEV or BSV. This approach is supported by the simulation results. In such situations a rather poor effectiveness of the PEV or BSV component on its own may become very substantial depending on the transmission intensity. So far we simulated combinations with matched durations of efficacy, however it may well be the case that MSTBVs have only very short-term effects because of an absence of natural boosting [21], so there is a need also to simulate combinations with high efficacy but short half-life MSTBV.

A number of the mass vaccination scenarios predict local elimination of the parasite. However, malaria is much easier to eliminate in computer simulations than in reality. This is particularly the case because transmission in nature is highly heterogeneous and this would be particularly important in low transmission settings [22], [23] so an adequate model for transmission heterogeneity would be essential for making useful predictions concerning local elimination. Our models currently allow for some heterogeneity in host response to infection and in infectiousness [24], [25] but not in susceptibility to infection. Furthermore, our simulations do not consider the effect of imported infections, which would have to be modelled taking into consideration the effectiveness of surveillance and heterogeneities in epidemiological receptivity.

Interestingly, we found that PEV vaccines in which the effect is concentrated in some individuals are more likely to achieve elimination. This arises because of the convex shape of the effectiveness vs initial efficacy curve (e.g. figure S1a–c) which implies, following Jensen's inequality [26] that variation in the efficacy will increase the average effectiveness. By an analogous argument we expect that heterogeneity in the vaccine half-life reduces effectiveness (see concave curve in figure S1d–f).

The simulations we present here should have implications for vaccine developers concerning the definition of minimal requirements for malaria vaccines to be used in public health. A PEV or BSV with a half-life of efficacy of less than 2–3 years will be of limited value and assessment of duration of protection is of great importance. Unfortunately Phase II trials are generally not designed to estimate duration. Since incidence declines steeply with age in young children, claims that efficacy is sustained in extended follow-ups [27], [28] are based on little data. Conversely, heterogeneities between individuals in susceptibility or vaccine ‘take’ lead to violation of proportional hazard assumptions [29] which appear as waning in apparent efficacy [18].

In addition to possible effects on transmission, vaccine developers would also be interested in determining whether substantial herd immunity effects are likely, and thus clinical development plans need to evaluate effects on transmission to the vector. We have found that, as could be expected, substantial transmission impact is generally achieved only if EPI delivery is supplemented by mass campaigns. Developers thus need to consider how vaccines are to be deployed. Probably, an important criterion for whether vaccines can be relatively easily deployed widely outside EPI is whether protection can be achieved with only 1 or 2 doses. In this respect, further analysis needs to focus on scenarios that are aligned with realistic distribution systems, using field data to identify realistic correlations between vaccination at successive rounds, rather than assuming these to be independent, and to assess what are feasible intervals between rounds.

Ultimately, malaria vaccines will be deployed as part of integrated control strategies. We thus plan further analyses to explore the interactions of vaccination with other malaria control interventions and the implications for resource allocation and management within the health system.

## Materials and Methods

### Epidemiological Model

We base our simulations of vaccines on our previously described model for the natural history and epidemiology of *P. falciparum* malaria [12]. This model uses an underlying within-host model based on detailed studies of the course of parasite densities in patients, who were treated for neurosyphilis in the 1950s with inoculations of malaria parasites. Morbidity, mortality, and transmission to the mosquito vector are treated as stochastic events with disease incidence related to the simulated parasite densities, and human demography is simulated with an algorithm that maintains the age structure of a typical rural African population.

For the present simulations we have recalibrated the model, using a genetic algorithm to parameterise it to 61 field scenarios from sub-Saharan Africa, comprising data on seasonality, age-patterns of infection, parasite density, clinical episodes, severe malaria and mortality[13]. The optimisation process made use of the Berkeley Open Infrastructure for Network Computing (BOINC; http://boinc.berkeley.edu/) which enables volunteer members of the public to run simulations of the field scenarios via links at www.malariacontrol.net, allowing parallel processing of many different computer intensive tasks. In addition to several thousand iterations for fitting, we make use of volunteer computing for over 32000 iterations/scenarios to produce the simulation results in this paper.

### Case management model

The simulations of the effects of vaccine interventions use a case management model, including both formal and informal treatment, based on that of a previous study to simulate existing case management in Tanzania [16]. To align our models with recent policy changes we modify this model to assume Artemisinin-based Combination Therapy (ACT) to be the first treatment for uncomplicated malaria while keeping with our previous model calibration that implied 4% of all fever attacks lead to official care for malaria. This change has implications, in terms of reduced rates of severe disease, sequelae and death, and also has an impact on transmission intensity. For our reference case, the model assumes that the ACT has a cure rate of 85%, which applies to 90% of patients that comply with the treatment schedule, and no effect for non-compliers [17]. Equity and heterogeneities in health systems are also important and are topics of later investigations, but beyond the scope of this work.

### Simulation of vaccines

Each simulated vaccine is characterised by an average initial efficacy, which is reached after completion of a vaccination schedule of 3 doses and thereafter decays exponentially. For the reference vaccine initial efficacy 52% after the third dose we assume efficacies for dose 1 and 2 used previously, namely 40% and 46% respectively [14]. For all other scenarios we assume the protective efficacy to increase linearly with the dose number, so that the efficacy after one dose is one third of the assumed maximum efficacy. To allow for heterogeneity in individuals' response to vaccination, we assign initial values for efficacy, which are drawn from a beta-distribution [14]. We quantify the degree of variation in response by the parameter *b* of the beta distribution and refer to this as the homogeneity parameter, which takes a high value when the vaccine effect is distributed evenly, and conversely a low value when the effect is concentrated in some individuals. All simulated vaccines are delivered at the pre-specified ages, but we consider a range of coverage values for vaccination (Table 1) to allow for individuals who do not complete the full course of vaccination.

**Table 1:** Summary of vaccination scenarios investigated

Vaccine combinations:	PEV (Pre-erythrocytic vaccine)
	BSV (Blood-stage vaccine)
	PEV+BSV
	PEV+TBV (Mosquito-stage transmission-blocking vaccine)
	BSV+TBV
	PEV+BSV+TBV
Delivery modality	EPI (1,2,3 Months)
	EPI with booster 1,2,3,4 years after the last EPI dose,
	EPI+Campaign: Mass vaccination 3 doses at start of intervention period, then 1 dose at 5,10,15 years.
Vaccine coverage	EPI: 89% 3rd dose; 95% 1st dose
	EPI with booster: 99% of previously vaccinated
	EPI+Campaigns: varying levels of coverage from 0% to 95% are considered.
Initial protected efficacies after dose 3	0.1 to 1 (stepsize 0.1) (reference **0.5**)
Half live of protective efficacy	0.5, 1, 1.5, 2, 2.5, 5, **10**, 20, no decay (years)
Between host variation in initial protective efficacy*	*b = * 0.01, **10**, 100000
Transmission intensity	EIR (infectious bites per annum) = 5.25, 10.5, **21**, 42, 84, 168

* parameter *b*

Figures in bold represent the value used for the reference scenario

#### (i) Pre-erythrocytic vaccines (PEV)

We assume pre-erythrocytic vaccines lead to a reduction in the proportion of sporozoite inocula that lead to blood stage infection, where the efficacy is equal to the proportional reduction in incidence of infection. This model is justified by analysis of the effects recorded in trials of the RTS,S vaccine [18].

#### (ii) Blood stage vaccines (BSV)

The immediate effect of a blood stage vaccine is assumed to be reduction in parasite density levels at each time step, where efficacy is equal to the proportional reduction.

#### (iii) Mosquito stage transmission blocking vaccines (MSTBV)

We model mosquito-stage transmission blocking vaccines by defining the efficacy to be the proportion by which the probability that a mosquito becomes infected from any one feed is reduced. We assume the efficacy of MSTBV to be proportional to the number of doses. This if the initial efficacy after the third dose is 95%, for first and second doses we assume initial efficacies of 32% and 63%, respectively.

#### (iv) Combination vaccines

We consider combination vaccines of PEV with MSTBV, BSV with MSTBV, BSV with PEV and also a combination of all three. In each case we assume PEV and BSV to be matched in the initial efficacy and in rate of decay. Since it is unlikely that MSTBV with only moderate efficacy will be developed, we consider combinations of PEV, BSV and of PEV-BSV with high efficacy MSTBV, and thus assume an MSTBV initial efficacy after the third dose of 95%. The rate of decay of MSTBV is matched to that of the other vaccine components.

### Vaccine delivery modalities

We model three delivery modalities:

#### (i) EPI

The first is the delivery through the EPI according to the usual DTP3 schedule (children 1, 2, 3 months of age).

#### (ii) EPI with booster

In this delivery modality, booster doses are added to the normal EPI schedule with booster doses at 1,2,3,4 years after the last EPI schedule. We assume that the effect of a booster dose of vaccine is to restore the protective efficacy to that achieved after the 3^rd^ dose in the same individual.

#### (iii) Mass vaccination with EPI catch-up

The third delivery modality combines the delivery of the vaccine to infants through EPI and a population-wide mass vaccination campaign with three doses at the beginning of the intervention period followed by additional mass vaccinations with a single dose after 5, 10, and 15 years. The protective efficacy of the vaccine is assumed to increase linearly up to dose 3. Additional doses restore the efficacy to that achieved at dose 3.

### Vaccine coverage

Under delivery modality (i), the vaccination coverage is as detailed in Table 1. This corresponds to that used in our previous models [14], in which the coverage of full vaccination is that reported in Tanzania for three doses of diphtheria tetanus pertussis–hepatitis B (DTP-HBV) vaccine in the year 2003, which was 95%, for the first dose and 89% for the third. Under delivery modality (ii) we assume that only those that receive the third EPI dose receive a booster dose. Coverage for booster doses is 99% of those that did not miss any of the previous vaccine doses. It is unlikely that those that miss an EPI dose will receive booster doses, and if included in the booster regime, it is unlikely further benefit would be predicted since coverage of the third EPI dose is relatively high (89%) and booster dosage coverage is very high (99%). For delivery modality (iii) we investigate different levels of coverage, ranging from 0% to 95%. We assume that in the initial campaign the percentage of the population covered at each of the three doses is the same and that after 5, 10, and 15 years, the same percentage of the population (all ages), but not necessarily the same individuals, receives a single dose.

### Measurement of effects of vaccination programs

The simulated scenarios cover all three vaccine types, and the three combinations, delivered through the three modalities at a range of coverage levels (Table 1). We also consider 6 different transmission intensities (Table 1). For each vaccine and combination, for each delivery strategy, coverage level and transmission intensity, we start from a reference set of assumptions and vary one of the efficacy parameters (initial efficacy, half-life and heterogeneity parameter), at a time. The parameters of Table 1 were chosen to consider a wide range of vaccine profiles, and thus to examine the effect of varying elements of half-life, efficacy and heterogeneity in response. The effect of each of these variations is evaluated by simulating the malaria dynamics in a population of 100,000 people over a 20 year period. Each simulation is repeated 3 times using different seeds to initialize the random number generator, and each of these simulations is compared with an independent simulation of a control population with no vaccine, but with the same human demography, baseline transmission setting, and health system. In general, variation between seeds in results is small. Measures of variation for predictions for different seed values are available on request.

### Measures of health gains

The main analyses consider the aggregated effects over the first 10 years of the vaccination program. We consider the effect of each vaccine on simulated values of a standard set of epidemiological outcomes in the whole population (not just those vaccinated): the number of uncomplicated malaria episodes, the number of severe malaria episodes and the number of deaths caused by malaria. For each of these outcomes we compute the number of events averted per 1000 person-years by comparing the vaccine simulation with the corresponding control simulation. We define the effectiveness as the proportion of events of each type that are averted. In addition, at each time point of the 20 years of the simulation we consider the proportion of mosquitoes that become infected at each feed as a measure of the level of transmission. We present predictions via plots of outcomes or estimates of average effectiveness for particular scenarios (Table S1, Supplementary material). Residual stochastic variation is small and consequently we do not present statistical significant tests.

## Supporting Information

Figure S1 Effect of initial efficacy (a–c), vaccine half-life (d–f) and degree of heterogeneity (g–i) on the effectiveness of PEV for the reference transmission setting of EIR 21. Results obtained assuming vaccine efficacy of 52%, a vaccine half-life of 10 years and homogeneity value of 10, unless the values are varied along the x-axis. Vaccines are distributed via EPI (circles), EPI with boosters (*) and EPI with 70% mass vaccination (squares).
(1.04 MB TIF)

Figure S2 Effect of initial efficacy on effectiveness of PEV for different transmission settings delivered via EPI with mass vaccination for 0% (a–c), 10% (d–f), 30% (g–i), 50% (j–l),7 0% (m–o) and 90% (p–r) coverage. Results obtained assuming a vaccine half-life of 10 years and homogeneity value of 10.
(1.46 MB TIF)

Figure S3 Effect of initial efficacy on effectiveness of BSV for different transmission settings delivered via EPI (a–c), EPI with boosters (d–f) and EPI with 70% mass vaccination (g–i). Results obtained assuming a vaccine half-life of 10 years and homogeneity value of 10.
(1.16 MB TIF)

Figure S4 Effect of initial efficacy on effectiveness of all vaccines for different transmission settings delivered via EPI and boosters (BSV (a–c), BSV/TBV (d–f), PEV (g–i), PEV/TBV (j–l), BSV/PEV (m–o) and BSV/TBV (p–r)). Results obtained assuming a vaccine half-life of 10 years and homogeneity value of 10.
(1.34 MB TIF)

Figure S5 Effect of vaccine half-life on effectiveness of all vaccines for different transmission settings delivered via EPI (BSV (a–c), BSV/TBV (d–f), PEV (g–i), PEV/TBV (j–l), BSV/PEV (m–o) and BSV/TBV (p–r)). Results obtained assuming an initial vaccine efficacy of 52% and homogeneity value of 10.
(1.14 MB TIF)

Figure S6 Effect of vaccine half-life on effectiveness of all vaccines for different transmission settings delivered via EPI with 70% mass vaccination (BSV (a–c), BSV/TBV (d–f), PEV (g–i), PEV/TBV (j–l), BSV/PEV (m–o) and BSV/TBV (p–r)). Results obtained assuming an initial vaccine efficacy of 52% and homogeneity value of 10.
(1.36 MB TIF)

Figure S7 Effect of the degree of heterogeneity on effectiveness of all vaccines for different transmission settings delivered via EPI (BSV (a–c), BSV/TBV (d–f), PEV (g–i), PEV/TBV (j–l), BSV/PEV (m–o) and BSV/TBV (p–r)). Results obtained assuming a vaccine half-life of 10 years and homogeneity value of 10.
(1.14 MB TIF)

Figure S8 Effect of initial efficacy on effectiveness of vaccine combinations with MSTBV for different transmission settings delivered via EPI with mass vaccination for 0% (a–c), 10% (d–f), 30% (g–i), 50% (j–l),7 0% (m–o) and 90% (p–r) coverage. Results obtained assuming a vaccine half-life of 10 years and homogeneity value of 10.
(1.62 MB TIF)

Table S1 Effectiveness (%) of each vaccine or combination over 10 years
(0.13 MB DOC)
