# Supplementary figures and images for: What Should Vaccine Developers Ask? Simulation of the Effectiveness of Malaria Vaccines
Source: PLoS One. 2008 Sep 11;3(9):e3193. doi: 10.1371/journal.pone.0003193 (PMC2527129; doi:10.1371/journal.pone.0003193)

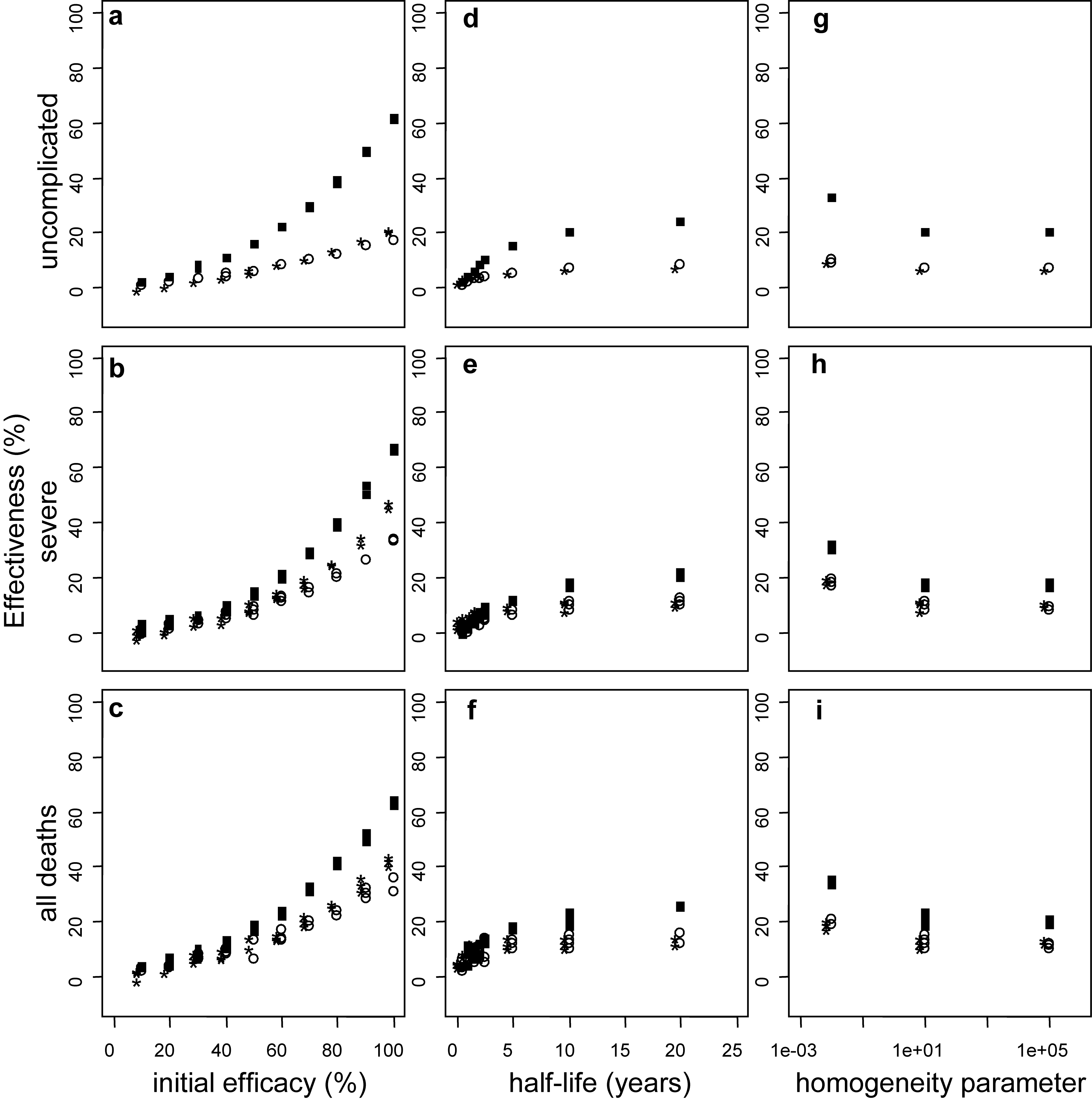

Supplement: Figure S1 — Effect of initial efficacy (a–c), vaccine half-life (d–f) and degree of heterogeneity (g–i) on the effectiveness of PEV for the reference transmission setting of EIR 21. Results obtained assuming vaccine efficacy of 52%, a vaccine half-life of 10 years and homogeneity value of 10, unless the values are varied along the x-axis. Vaccines are distributed via EPI (circles), EPI with boosters (*) and EPI with 70% mass vaccination (squares). (1.04 MB TIF) [file pone.0003193.s001.tif]

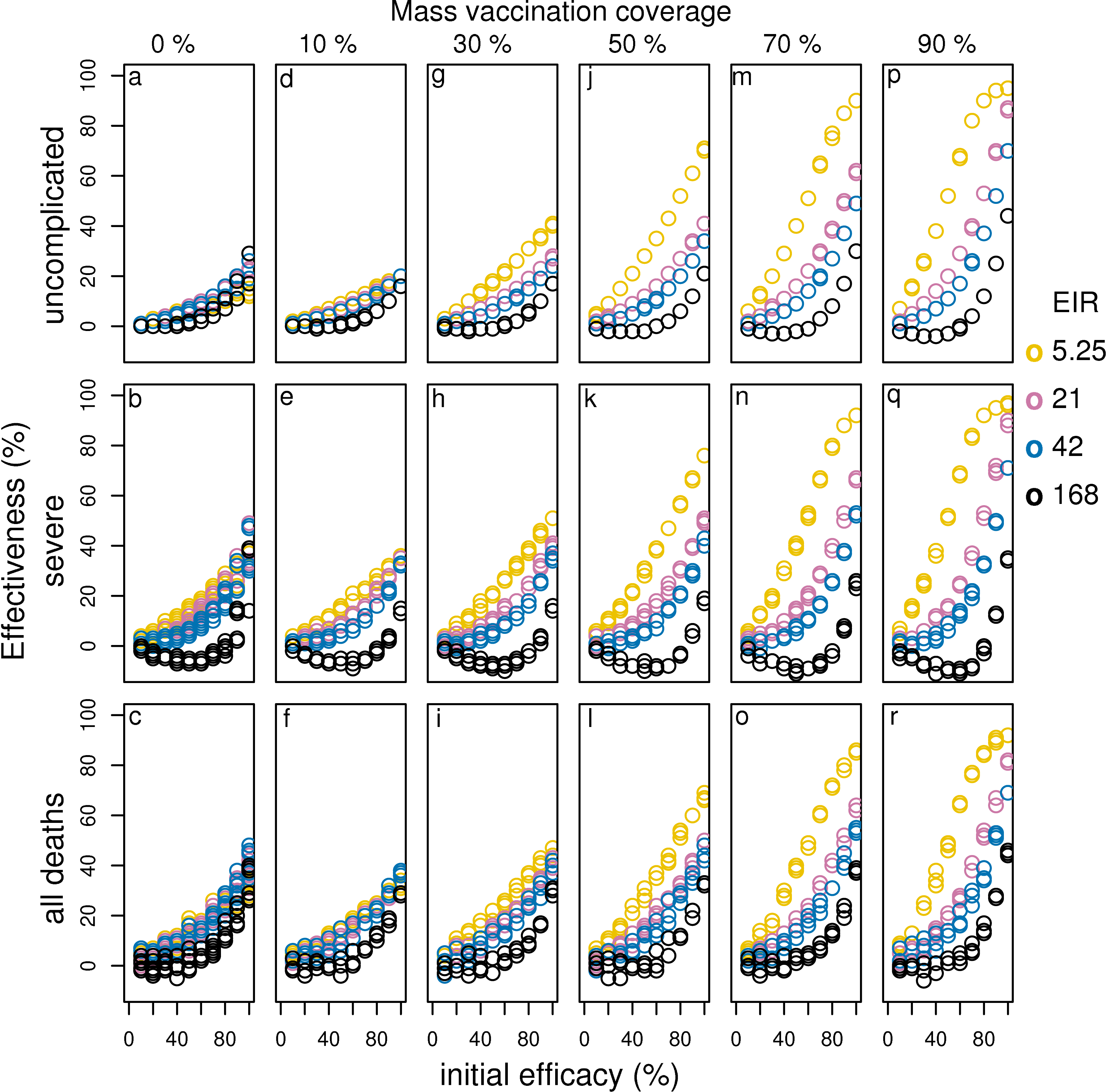

Supplement: Figure S2 — Effect of initial efficacy on effectiveness of PEV for different transmission settings delivered via EPI with mass vaccination for 0% (a–c), 10% (d–f), 30% (g–i), 50% (j–l),7 0% (m–o) and 90% (p–r) coverage. Results obtained assuming a vaccine half-life of 10 years and homogeneity value of 10. (1.46 MB TIF) [file pone.0003193.s002.tif]

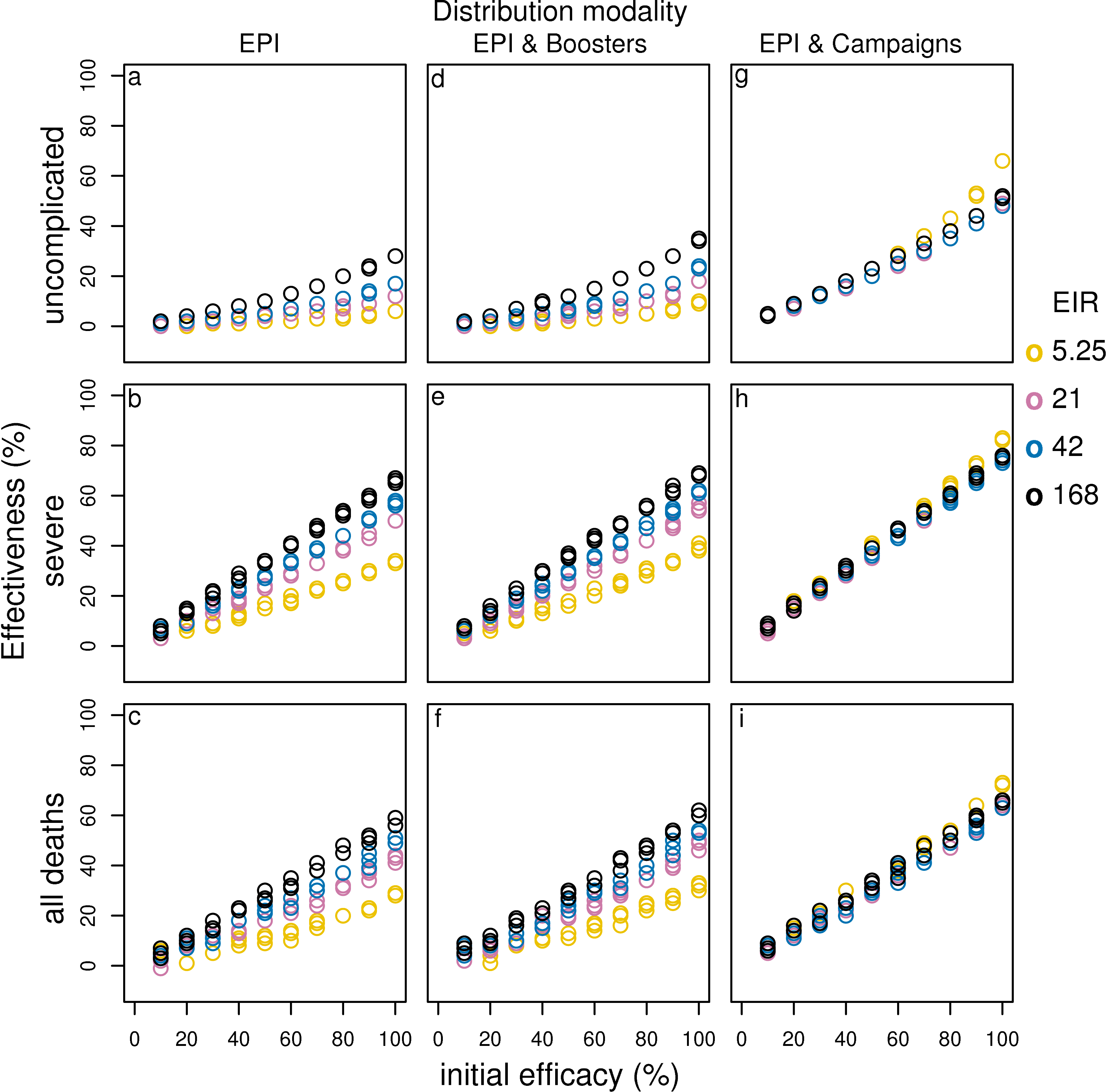

Supplement: Figure S3 — Effect of initial efficacy on effectiveness of BSV for different transmission settings delivered via EPI (a–c), EPI with boosters (d–f) and EPI with 70% mass vaccination (g–i). Results obtained assuming a vaccine half-life of 10 years and homogeneity value of 10. (1.16 MB TIF) [file pone.0003193.s003.tif]

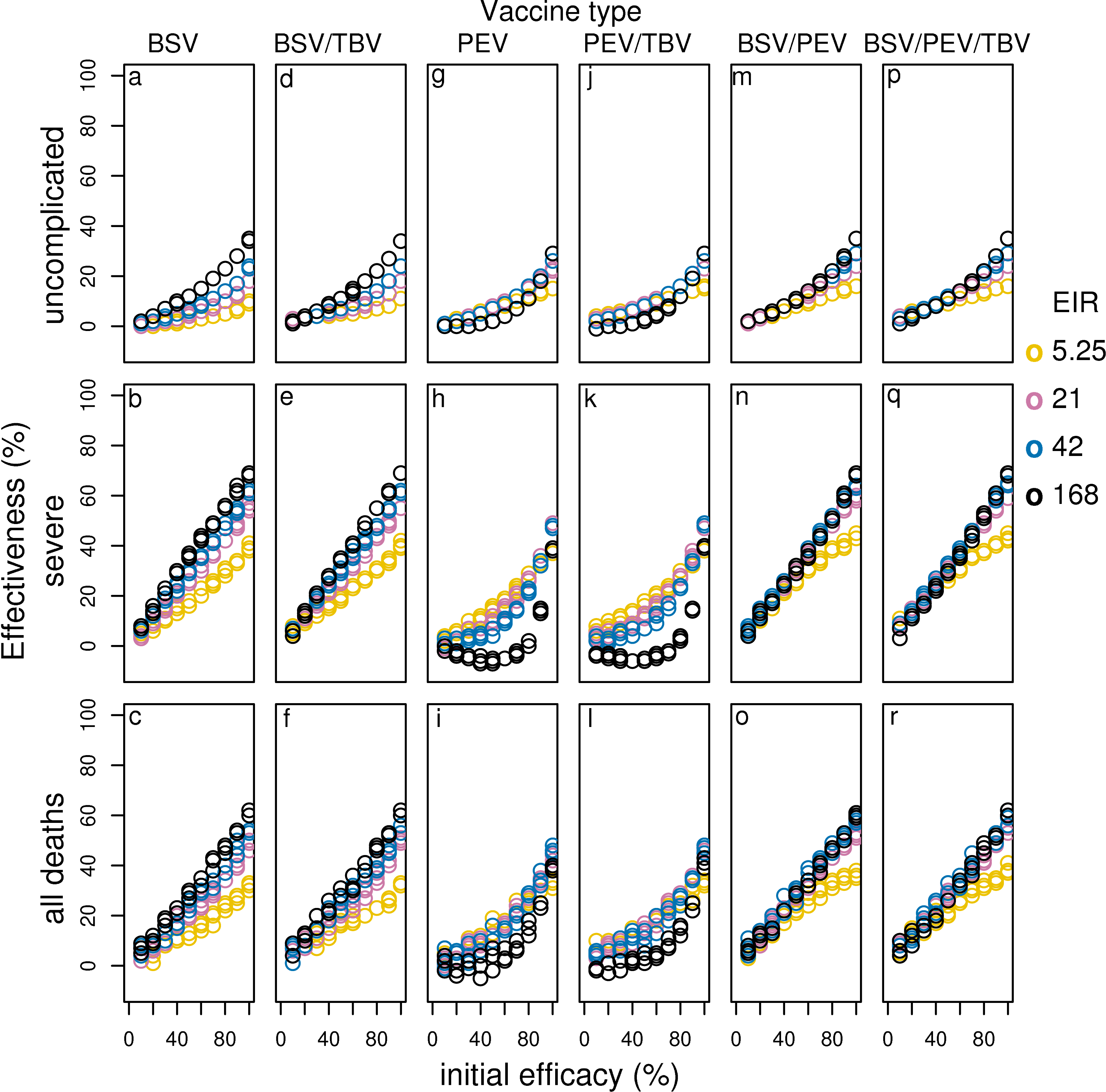

Supplement: Figure S4 — Effect of initial efficacy on effectiveness of all vaccines for different transmission settings delivered via EPI and boosters (BSV (a–c), BSV/TBV (d–f), PEV (g–i), PEV/TBV (j–l), BSV/PEV (m–o) and BSV/TBV (p–r)). Results obtained assuming a vaccine half-life of 10 years and homogeneity value of 10. (1.34 MB TIF) [file pone.0003193.s004.tif]

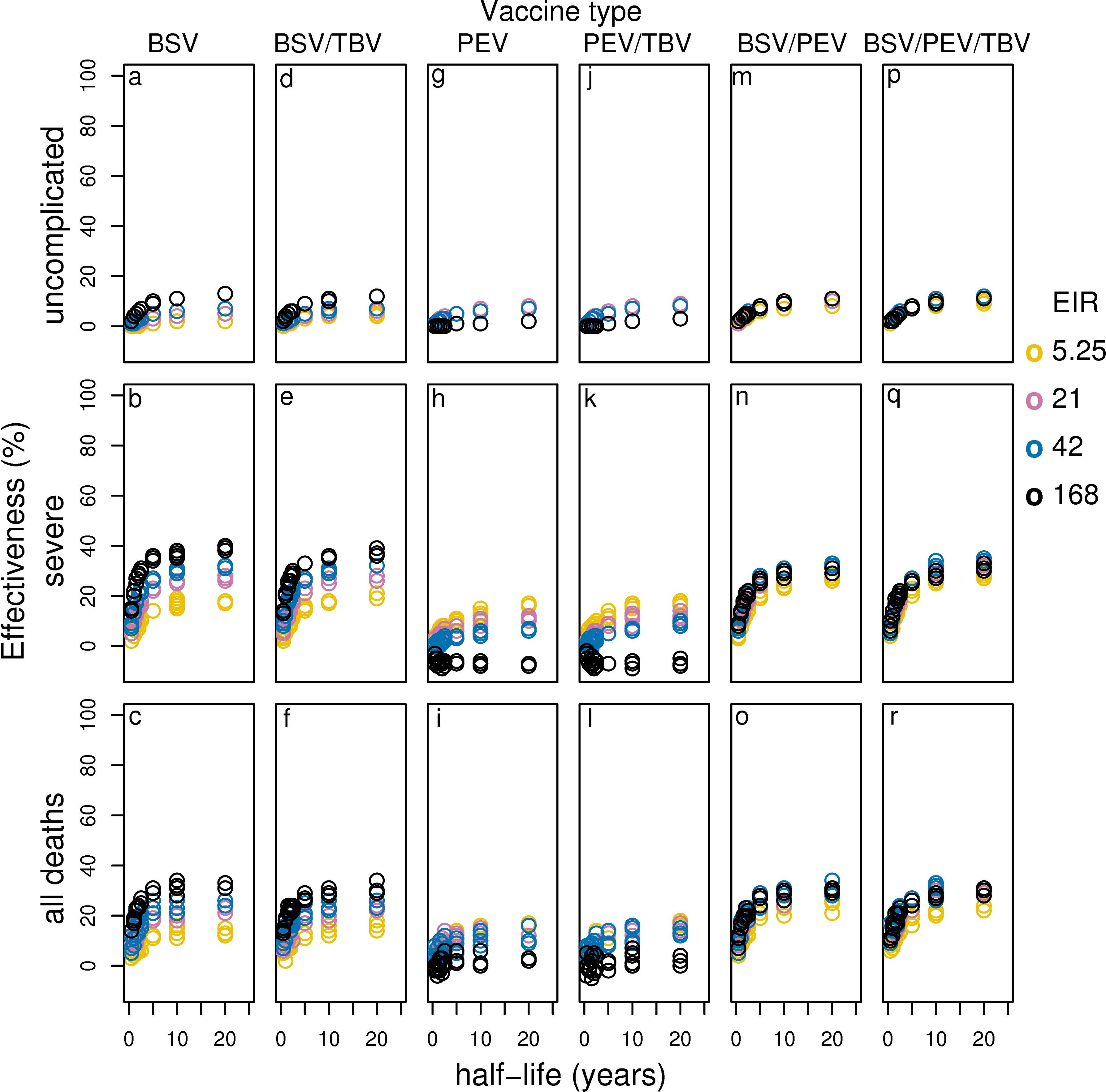

Supplement: Figure S5 — Effect of vaccine half-life on effectiveness of all vaccines for different transmission settings delivered via EPI (BSV (a–c), BSV/TBV (d–f), PEV (g–i), PEV/TBV (j–l), BSV/PEV (m–o) and BSV/TBV (p–r)). Results obtained assuming an initial vaccine efficacy of 52% and homogeneity value of 10. (1.14 MB TIF) [file pone.0003193.s005.tif]

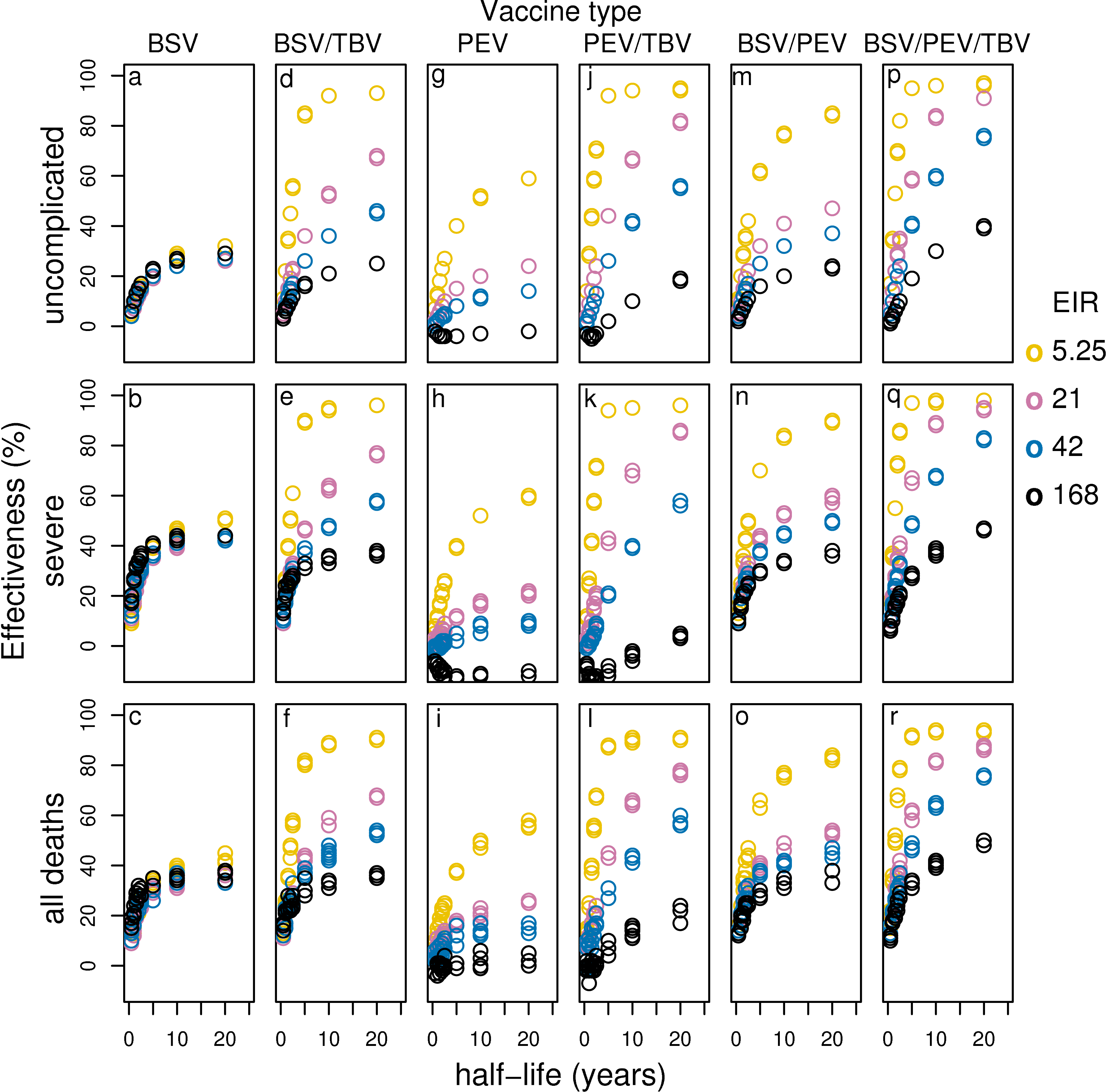

Supplement: Figure S6 — Effect of vaccine half-life on effectiveness of all vaccines for different transmission settings delivered via EPI with 70% mass vaccination (BSV (a–c), BSV/TBV (d–f), PEV (g–i), PEV/TBV (j–l), BSV/PEV (m–o) and BSV/TBV (p–r)). Results obtained assuming an initial vaccine efficacy of 52% and homogeneity value of 10. (1.36 MB TIF) [file pone.0003193.s006.tif]

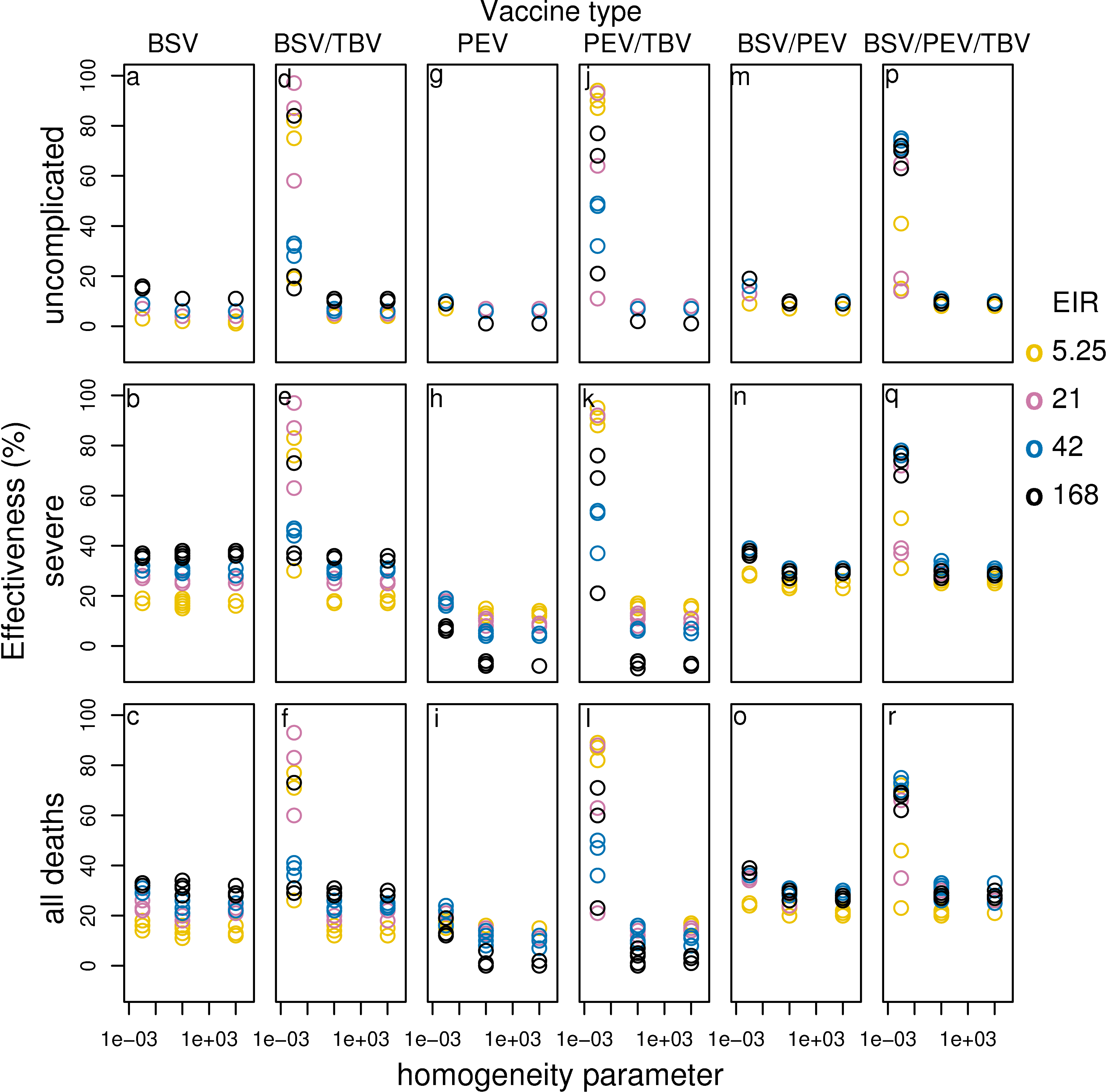

Supplement: Figure S7 — Effect of the degree of heterogeneity on effectiveness of all vaccines for different transmission settings delivered via EPI (BSV (a–c), BSV/TBV (d–f), PEV (g–i), PEV/TBV (j–l), BSV/PEV (m–o) and BSV/TBV (p–r)). Results obtained assuming a vaccine half-life of 10 years and homogeneity value of 10. (1.14 MB TIF) [file pone.0003193.s007.tif]

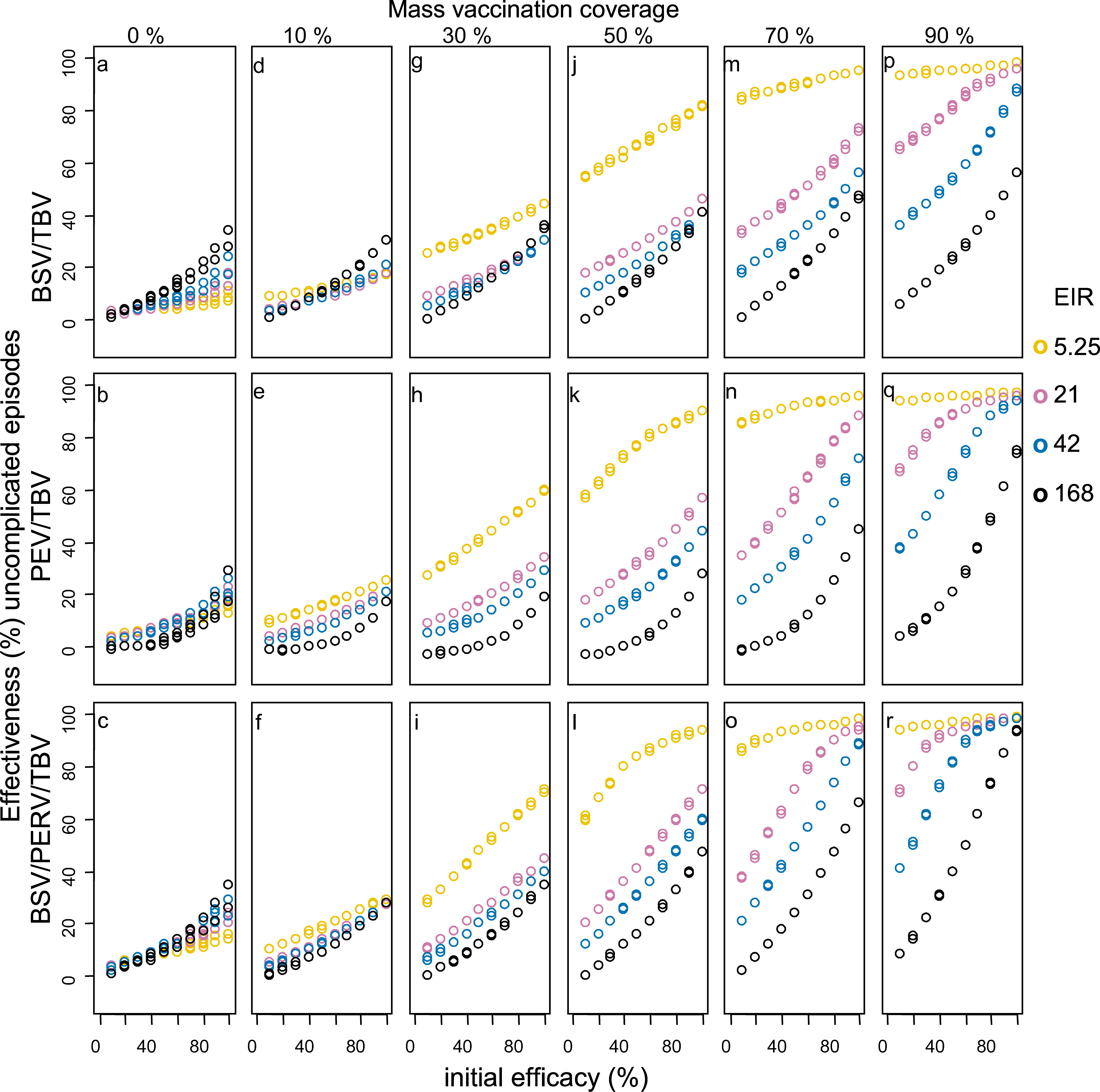

Supplement: Figure S8 — Effect of initial efficacy on effectiveness of vaccine combinations with MSTBV for different transmission settings delivered via EPI with mass vaccination for 0% (a–c), 10% (d–f), 30% (g–i), 50% (j–l),7 0% (m–o) and 90% (p–r) coverage. Results obtained assuming a vaccine half-life of 10 years and homogeneity value of 10. (1.62 MB TIF) [file pone.0003193.s008.tif]
