# Supplementary material for: What Should Vaccine Developers Ask? Simulation of the Effectiveness of Malaria Vaccines
Source: PLoS One. 2008 Sep 11;3(9):e3193. doi: 10.1371/journal.pone.0003193 (PMC2527129; doi:10.1371/journal.pone.0003193)
Supplement: Table S1 — Effectiveness (%) of each vaccine or combination over 10 years (0.13 MB DOC) [file pone.0003193.s009.doc]

**Table S1 – Effectiveness (%) of each vaccine or combination over 10 years**

|  |  | **Uncomplicated** | | | | | **Severe episodes** | | | | | **All deaths** | | | | |
| --- | --- | --- | --- | --- | --- | --- | --- | --- | --- | --- | --- | --- | --- | --- | --- | --- |
|  | **EIR** | **EPI** | **EPI booster** | **EPI 70% campaign** | **EPI 50% campaign** | **EPI 30% campaign** | **EPI** | **EPI booster** | **EPI 70% campaign** | **EPI 50% campaign** | **EPI 30% campaign** | **EPI** | **EPI booster** | **EPI 70% campaign** | **EPI 50% campaign** | **EPI 30% campaign** |
| **BSV** | 5.25 | 2.0 | 2.0 | 28.8 | 24.0 | 17.3 | 16.7 | 18.5 | 45.8 | 41.5 | 33.8 | 13.5 | 13.3 | 38.5 | 35.0 | 27.2 |
|  | 21 | 4.0 | 5.0 | 24.0 | 20.7 | 16.0 | 25.5 | 27.8 | 40.5 | 38.2 | 34.8 | 19.3 | 23.0 | 33.3 | 31.0 | 27.7 |
|  | 84 | 8.5 | 10.0 | 25.0 | 22.3 | 18.3 | 33.5 | 35.0 | 41.5 | 40.8 | 38.2 | 27.7 | 28.7 | 34.3 | 32.5 | 32.0 |
|  | 168 | 11.0 | 13.3 | 26.2 | 24.0 | 20.3 | 36.5 | 38.3 | 43.3 | 42.3 | 40.3 | 31.2 | 31.0 | 35.3 | 35.5 | 33.5 |
| **BSV TBV** | 5.25 | 4.0 | 5.0 | 92.0 | 74.3 | 39.8 | 17.7 | 20.3 | 94.7 | 81.7 | 51.8 | 14.7 | 16.2 | 88.3 | 72.7 | 45.3 |
|  | 21 | 5.0 | 6.0 | 52.3 | 33.0 | 22.0 | 25.7 | 28.7 | 63.0 | 46.5 | 37.2 | 20.3 | 22.7 | 57.0 | 42.0 | 32.0 |
|  | 84 | 8.3 | 10.0 | 27.0 | 22.0 | 17.7 | 33.5 | 34.7 | 39.7 | 37.7 | 36.3 | 27.5 | 28.2 | 37.3 | 33.2 | 31.8 |
|  | 168 | 10.8 | 12.2 | 21.0 | 19.0 | 17.0 | 35.8 | 37.3 | 34.8 | 35.3 | 37.3 | 29.5 | 31.5 | 33.0 | 33.0 | 31.7 |
| **PEV** | 5.25 | 6.0 | 7.0 | 51.5 | 41.8 | 29.0 | 13.5 | 16.0 | 52.0 | 42.3 | 31.2 | 13.8 | 15.0 | 49.3 | 39.5 | 28.5 |
|  | 21 | 7.0 | 8.0 | 20.0 | 17.0 | 13.0 | 9.8 | 11.2 | 17.2 | 14.3 | 11.7 | 12.2 | 13.0 | 21.0 | 18.0 | 15.7 |
|  | 84 | 4.2 | 5.8 | 4.0 | 3.7 | 3.3 | -0.2 | 0.5 | -2.7 | -1.7 | -2.3 | 6.7 | 5.7 | 6.3 | 7.3 | 6.3 |
|  | 168 | 1.0 | 2.0 | -3.0 | -3.0 | -2.0 | -7.0 | -6.0 | -11.3 | -10.7 | -9.3 | 2.3 | 4.5 | 1.0 | 3.3 | 1.7 |
| **PEV TBV** | 5.25 | 8.0 | 9.0 | 94.0 | 86.3 | 53.8 | 15.8 | 17.2 | 95.0 | 88.8 | 56.7 | 15.5 | 16.7 | 89.7 | 80.3 | 51.5 |
|  | 21 | 8.0 | 9.3 | 66.3 | 39.3 | 22.3 | 10.8 | 13.3 | 68.7 | 39.2 | 22.3 | 13.7 | 16.2 | 65.0 | 40.8 | 26.7 |
|  | 84 | 5.0 | 6.5 | 24.0 | 13.0 | 7.0 | -0.3 | 2.3 | 16.5 | 5.2 | 0.7 | 7.2 | 8.7 | 25.0 | 17.7 | 10.7 |
|  | 168 | 2.0 | 3.0 | 10.0 | 3.0 | 0.0 | -7.2 | -6.7 | -3.2 | -8.8 | -8.3 | 3.5 | 2.7 | 13.3 | 6.8 | 3.3 |
| **BSV PEV** | 5.25 | 7.0 | 8.0 | 76.7 | 63.0 | 43.7 | 24.2 | 27.0 | 83.2 | 72.2 | 54.8 | 21.7 | 22.0 | 76.0 | 65.8 | 47.8 |
|  | 21 | 9.0 | 11.0 | 41.0 | 34.0 | 26.0 | 29.5 | 33.0 | 52.3 | 47.0 | 41.0 | 26.7 | 29.0 | 48.0 | 43.3 | 38.3 |
|  | 84 | 10.0 | 12.0 | 25.7 | 22.3 | 18.2 | 29.7 | 33.2 | 38.5 | 37.0 | 34.3 | 26.7 | 29.0 | 37.2 | 35.2 | 32.0 |
|  | 168 | 9.2 | 11.3 | 20.0 | 18.0 | 15.0 | 28.8 | 31.3 | 33.7 | 32.7 | 31.0 | 28.2 | 29.7 | 33.0 | 32.0 | 30.3 |
| **BSV PEV TBV** | 5.25 | 8.3 | 10.0 | 96.0 | 91.7 | 66.0 | 25.3 | 28.3 | 97.8 | 94.7 | 74.3 | 21.0 | 23.7 | 93.5 | 88.7 | 66.7 |
|  | 21 | 10.0 | 12.0 | 83.7 | 54.3 | 33.8 | 30.0 | 34.0 | 88.7 | 64.7 | 47.3 | 28.7 | 31.2 | 81.5 | 60.3 | 42.7 |
|  | 84 | 10.2 | 12.0 | 42.0 | 29.3 | 21.0 | 30.3 | 33.0 | 50.7 | 40.7 | 35.0 | 27.7 | 31.2 | 49.7 | 41.3 | 34.3 |
|  | 168 | 9.5 | 11.3 | 30.0 | 21.0 | 16.0 | 28.5 | 31.5 | 37.7 | 33.0 | 30.3 | 28.0 | 30.3 | 40.0 | 35.7 | 31.0 |

Each vaccine is assumed to have an initial efficacy of 52%, half-life of 10 years and a homogeneity factor of 10. The results shown are averages of three simulation runs.
